# Elevated Cardiac Troponins in Setting of Systemic Inflammatory Response Syndrome, Sepsis, and Septic Shock

**DOI:** 10.1155/2013/723435

**Published:** 2013-04-11

**Authors:** Nasir Hussain

**Affiliations:** Saint Joseph Hospital, Presence Health, Department of Internal Medicine, 2900 North Lake Shore Drive, Chicago, IL 60657, USA

## Abstract

Elevation of cardiac troponins and creatinine kinase is frequently observed in setting of systemic inflammatory response syndrome (SIRS), sepsis, or septic shock. Underlying pathophysiologic mechanism for such troponin leak, its clinical significance, and what different could be done in such settings remain elusive. In this paper we have briefly overviewed the proposed pathogenic mechanisms for SIRS, sepsis, or septic shock-related troponin elevation (SRTE) and have provided brief overview on its clinical significance. Upon review of the relevant literature we found that majority of patients with the SRTE with no prior history of coronary artery disease (CAD) upon testing are found not to have any CADs. We have also briefly discussed the possible pharmacologic agents and potential targets which are important from pathophysiologic and pharmacologic point of view that may alter the outcomes of SRTE-related myocardial depression in near future.

## 1. Introduction

Elevation of cardiac troponins and creatinine kinase (CK) is observed in 31%–80% of patients in setting of systemic inflammatory response syndrome (SIRS), sepsis, or septic shock (SIRS, sepsis, or septic shock related troponin elevations (SRTE)) [1–3]. Skeletal muscle ischemia due to sepsis-related hypotension explains the elevated CK [3]. Cardiac troponins are elevated only when there is an insult to cardiac myocytes; what causes this insult in the setting of sepsis is not known. Different theories have been hypothesized to explain the SRTE. From academic as well as from a clinical standpoint defining the etiopathogenesis of the SRTE and its significance will have important clinical implications. There are no consensus guidelines on how to approach patients with SRTE. 

Majority of SRTE patients without any prior history of coronary artery disease (CAD) on testing are found not to have any significant CADs [3–5] (Table 1). Troponin elevation in setting of sepsis has been proposed as a biomarker for underlying myocardial dysfunction [6]. Sepsis-related mortality has been reported to equal the mortality due to myocardial infarction [7, 8] and myocardial dysfunction has been shown to be a common complication in the setting of sepsis [7, 8].

The purpose of this paper is to briefly review three basic questions: what causes SRTE, what is its clinical significance and what different can be done in such cases?

## 2. What Is Cardiac Troponin?

Troponin is a complex of three regulatory proteins (troponin I, TnI, troponin C, TnC, troponin T, TnT) [6] and TnT binds to tropomyosin that lies in between the groves of actin [6], TnI binds to actin whereas TnC binds to calcium [6]. Troponin is integral to contractile mechanism of cardiac and skeletal muscles. Binding of calcium on TnC leads to a conformal change in TnI and thereby in tropomyosin which exposes myosin binding sites on actin leading to actin and myosin interaction and muscle contraction [6]. TnI and TnT of skeletal and cardiac muscles have different amino acid sequences; the same is not true for Tnc [6]. TnI is much more specific for detection of any damage to cardiac myocytes [6] as compared to TnT, and TnI levels do not increase in setting of renal failure [2]. 

## 3. What Causes the SRTE? Etiology and Pathogenesis of the SRTE

### 3.1. Demand and Supply Mismatch Theory

Most popular theory for explanation of SRTE has always been the demand and supply mismatch theory [3]. In the setting of sepsis, the cardiac metabolic requirements are high [3, 14, 15] and in order to meet these requirements an increase in the coronary blood flow is needed. Patients with underlying anemia and preexisting subclinical CAD may develop a mismatch ischemia [3] in this setting. It was always thought that sepsis-related hypotension causes a decrease in coronary perfusion pressure [3] thus leading to a decreased blood flow to cardiac myocytes and thereby leading to SRTE. However contrary to popular belief, Cunnion et al. and Dhainaut et al. [14, 15] showed that in setting of sepsis coronary blood flow actually increases which argues against the theory of demand and supply mismatch. However, recent experimental studies have shown that in setting of sepsis generalized or focal microvascular dysfunction [16–19] does occur that leads to myocardial ischemia and SRTE. Ischemia due to microvascular dysfunction is not a demand ischemia. Preexisting anemia, tachycardia, and high myocardial oxygen demand in setting of sepsis theoretically [6] may aggravate ischemia due to microvascular dysfunction. Autopsy studies have demonstrated presence of contraction band necrosis in setting of SRTE [6] (contract band necrosis is typically associated with ischemia) which suggests that myocardial ischemia may have a role in the pathogenesis of SRTE. 

### 3.2. Stress-Mediated SRTE

There are two storage forms of cardiac troponins: cytosolic and myofibril. Quantity of troponins in cytosol is 35 times lower than in myofibril [2]. It has been hypothesized that in a setting of stress, cytosolic troponins may leak and lead to a rise in blood levels even in the absence of any damage to myofibril [2]. This hypothesis could partially explain SRTE as there is evidence of myofibril ischemic damage in setting of sepsis as described previously. 

### 3.3. Direct Myocarditis and Role of Cytokines and Vasopressors in SRTE

**Figure 1:**
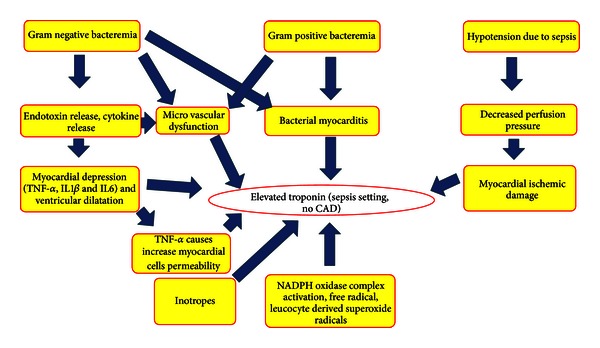
Pathogenic mechanisms of SRTE.

Bacterial myocarditis [3] leading to release of troponins in absence of CAD has also been suggested as a possible pathogenic mechanism for SRTE. Release of cytokines (IL1*β*, IL-6, and TNF*α*), nitric oxide, endotoxins [3], and activation of caspases (caspases 3) [20] in setting of a gram negative bacteremia and sepsis leading to myocardial depression and ventricular dilatation [3] is another theory to explain SRTE (Figure 1). Considering that, TNF*α* increases the permeability of endothelial cells to macromolecules and lower molecular weight solutes, researchers have proposed that a similar increase in permeability of myocardial cell membrane may be responsible for SRTE [3, 21]. Frequent use of inotropes in setting of sepsis may have an impact on SRTE [3, 6]. Troponin elevation in setting of SIRS, sepsis, or septic shock may act as a biomarker for underlying myocardial depression [3]. IL1*β*, IL-6, and TNF*α* are believed to play a central role in sepsis-mediated myocardial depression [22]. TNF*α*-mediated activation of neutral sphingomyelinase, suppression of nitric oxide and calcium transient pathways, modulation of intracellular proteases [22], effect on arachidonate metabolism, on protein kinases, oxygen-free radicals, nitric oxide, transcription of cytotoxic genes, nuclear regulatory factors, and ADP ribosylation are examples of a few mechanisms by which TNF*α* causes myocardial depression and SRTE [6].

### 3.4. Microthrombosis Theory

Altmann et al. [4] in a study demonstrated that no significant differences exist in coagulation parameters among SIRS, sepsis, and septic shock-related troponin positive or troponin negative subsets of patients. The previously mentioned finding suggests that thrombus related mechanisms are less likely, if any to play a role, in the pathogenesis of SRTE. 

### 3.5. Free Radicals and Superoxide Radicals Mediated SRTE

Activation of NADPH oxidase complexes and mitochondria (18) in setting of sepsis leads to a formation of free radicals. These free radicals along with leucocyte-derived superoxide radicals (19) are thought to cause myocardial cell damage and apoptosis leading to SRTE (19). 

### 3.6. Ventricular Wall Stress-Mediated SRTE

Increased cardiac filling pressures and increased wall stress in setting of sepsis have been proposed to activate intracellular signaling cascade leading to cardiac myocytes apoptosis [23], myocytes damage, and micronecrosis (21) leading to SRTE. Possible pathogenic mechanisms for SRTE are shown in Figure 1. 

Gram negative as well as gram positive bacterial [3] and fungal infections [4] have been shown to cause SRTE. Gram positive pathogens do not produce endotoxins which suggest that mechanisms other than release of endotoxins are responsible for SRTE [3]. Direct bacterial or fungal myocardial cell damage leading to an elevation in cardiac troponins theoretically seems more likely. Studies have shown that SRTE may occur irrespective of use of any inotropes [3] which suggests that inotropes may or may not play a role in the pathogenesis of SRTE.

## 4. The Clinical Significance of SRTE

On review of the literature we found that majority of patients with SIRS, sepsis, and septic shock-related deaths had positive cardiac troponins at time of death (Table 1). Thus it can be said that SRTE may be an indicator of worse underlying prognosis [2–4]. 

In setting of sepsis, heart undergoes different physiologic and metabolic changes which normalize within 7–10 days [14] in survivors. Physiologic changes include ventricular dilatation, depression of ejection fraction, and regional and global wall hypokinesia, as well as systolic and diastolic dysfunction [14]. Metabolic changes include increased lactate consumption, a decreased extraction of oxygen across the coronary circulation, maintenance of normal high-energy phosphate state, and increase in coronary blood flow [15]. The physiologic changes of the heart in setting of sepsis and their clinical significance along with the significance of SRTE will be discussed in detail.

SRTE has been proposed as a biomarker of underlying myocardial dysfunction (a major contributor to the worse outcomes in the setting of SRTE) [6] in setting of sepsis. Sepsis-mediated myocardial dysfunction results in reduced stroke volume either by systolic [6] or diastolic dysfunction [24] or combination of both [6]; generalized or regional hypokinesia of left ventricular wall may be seen in setting of sepsis [14]. Assessing systolic function on the basis of echocardiogram derived left ventricular ejection fraction (LVEF) in such settings may be misleading as the afterload is remarkably reduced [25] and LVEF may be inappropriately normal or near normal. Investigators have used different echocardiographic cutoffs to define myocardial dysfunction in setting of sepsis; for example, Fernandes et al. [9] used LVEF < 50% as a marker of myocardial dysfunction whereas Ver Elst et al. [10] used presence of an increased left ventricular end-diastolic diameter >60 mm, volume >120 cm^3^, and presence of regional and global hypokinesia and a left ventricular fractional area contraction (LVFAC) of <0.4 under inotropic support as diagnostic criteria for SRTE-related myocardial dysfunction. Fernandes et al. [9] compared TnI and echocardiogram derived left ventricular ejection fraction as biomarkers for underlying sepsis-related myocardial dysfunction in patients with no prior cardiac disease and they found that TnI was elevated in 6 out of 10 patients (total study population) whereas myocardial dysfunction was present only in 4 of 6 troponin positive patients on echocardiogram (myocardial dysfunction was defined as LVEF < 50%); remaining patients had neither TnI elevation nor any evidence of reduced LVEF; there was a mortality of 40%, and among nonsurvivors, 50% had normal LVEF on echocardiogram and 75% had TnI elevation (Table 1). In a study by Ver Elst et al. [10] transesophageal echocardiogram demonstrated left ventricular dysfunction in 78% of TnI positive patients but only in 2% of TnI negative patients. Results from previously described studies suggest that, in setting of sepsis, TnI may act as a better and sensitive biomarker for detection of myocardial dysfunction and associated worse prognosis as compared to echocardiogram. 

Contribution of right ventricle in sepsis-related myocardial dysfunction is not known, though similar sepsis-related physiologic changes are believed to affect both ventricles [15].

The concept of myocardial depression/dysfunction was for the first time described by Parker et al. [26], who performed serial radionuclide ventriculograms in 20 patients with septic shock, 7 of whom died during stay in the ICU, and survivors were observed to have depressed LVEF < 0.4 whereas none of the nonsurvivors had LVEF < 0.4; author proposed that nonsurvivors had a marked decrease in the systemic vascular resistance which resulted in normalization of LVEF among nonsurvivors. Very interesting data on cardiac hemodynamics was presented by Poelaert et al. [27] in a study where they used transesophageal echocardiography and invasive cardiac monitoring to assess the ventricular function in patients with septic shock. Poelaert et al. [27] classified patients into three groups: patients with normal LVFAC with normal transmitral and pulmonary flow (normal systolic and diastolic function), patients with normal LVFAC and abnormal pulmonary vein flow (isolated diastolic dysfunction), and patients with decreased LVFAC with abnormal transmitral and pulmonary vein flow pattern (diastolic dysfunction as a consequence of systolic function) and they described that patients in the latter group were significantly older and had higher mortality as compared to other groups; these findings were a contradiction to earlier findings by Parker et al. [26].

Better imaging techniques which could assess true systolic and diastolic dysfunction in setting of sepsis, could assess myocardial wall strain and perfusion may serve as a better biomarker for detection of myocardial dysfunction in setting of sepsis.

Different authors have used different cutoff values for TnT and TnI to define an elevation; in this review we have taken troponins positive or negative according to each author's criteria (Table 1). Further, different authors have used either TnI or TnT as a biomarker for SRTE-related myocardial dysfunction; for the purpose of this review we have considered both TnT and TnI together as marker of SRTE-related myocardial dysfunction/depression (Table 1). 

Studies have shown that presence of cardiovascular dysfunction in setting of sepsis is associated with a significant increase in mortality rate to 70%–90% as compared to 20% with no cardiovascular compromise [28]. On the other hand, Rudiger and Singer [29] noted that survivors of sepsis had a lower ejection fraction and a higher end-diastolic volume as compared to nonsurvivors. This may suggest that myocardial depression in setting of sepsis may have a protective role [29]. SRTE has been shown to associate with increased length of ICU stay [30] and with need for inotropic/vasopressor support [11]. It has been suggested that SRTE may represent a reversible insult to myocardium; in a study by Ver Elst et al. [10], no evidence of irreversible myocytes necrosis was found in autopsy cases of septic shock where there was a positive premortem troponin I. SRTE has been shown to associate with the severity of the disease as expressed by global scores such as acute physiology and chronic health evaluation (APACHE) II score or simplified acute physiology score II [10, 11]. 

Very few studies have considered the impact of preexisting risk factors on incidence of SRTE. Smith et al. in their study showed that diabetes was more prevalent in SRTE study population [31] and that preexisting hypertension or CAD did not affect the incidence of SRTE. Arlati et al. demonstrated that duration of hypotension during septic shock corresponded to a rise in cardiac troponins [12]. 

## 5. What Different Could Be Done in Setting of SRTE?

In the current practice, patients found to have elevated troponins in settings of SIRS, sepsis, or septic shock are either observed or undergo some form of noninvasive or invasive cardiac testing to rule out CAD. History of chest pain is difficult to obtain in most of SRTE patients as majority is intubated or sedated or too sick to communicate [30]. By review of the relevant literature we found that above 90% of such patient population with no prior history of CAD upon testing have no evidence of CAD (3, 4, 5). From this we could conclude that patients with isolated troponin elevation in absence of chest pain, chest tightness or other suggestive signs and symptoms of CAD with nondiagnostic ST T wave changes and no significant CAD risk factors may not need at least invasive procedure for diagnosis of CAD in acute setting. 

Acute coronary syndrome medications like aspirin, beta blockers, and ACE inhibitors in setting of SRTE have not been shown to improve prognosis [31]. 

Role of TNF*α* inhibitors [32–35] and IL1 receptor antagonist in setting of SRTE-related myocardial dysfunction is controversial [36, 37]. Similarly the role of cyclooxygenase inhibitors [38, 39] and endothelin receptor antagonists in setting of SRTE-related myocardial dysfunction has not been proven and is controversial [40]. Nitric oxide (NO) and microvascular dysfunction have been closely linked [41]; inhibition of NO represents a potential target for prevention of microvascular injury and thus SRTE [42]. Blockade of intercellular adhesion molecule-1 and vascular adhesion molecule-1 has shown promise in prevention of myocardial dysfunction in rats in setting of sepsis [43, 44]. Beta receptor antagonists [45] and statins [46] may also play a role in limiting myocardial dysfunction in septic cardiomyopathy. Beneficial role of activated protein C [47] and low-dose hydrocortisone in setting of sepsis has been documented [48]. Previously, Drotrecogin-*α* [49] was shown to reduce the incidence of SRTE in patients with severe sepsis and whether if the observed finding was a result of reduced microvascular dysfunction remains unknown. Recently, Drotrecogin-*α* was withdrawn from market due to lack of survival benefit in setting of septic shock. 

In conclusion, future prospective studies with a large patient population are needed to define the role of cardiac testing in the setting of SRTE and to define the exact pathogenesis of SRTE. Randomized controlled trials are needed to determine the optimal treatment strategy in septic patients with SRTE. Addition of cardiac troponins in sepsis protocol may help risk-stratify patients so that appropriate measures like echocardiograms and cardiac consults could be taken early in the course of the disease. 

## Figures and Tables

**Table 1:** 

Authors	Total population	Troponin positive	No CAD in troponin positive (%) patients	CAD in troponin positive (%)	CAD could not be determined in troponin positive	No CAD in troponin positive who underwent testing	Total deaths	Troponin positive patients deaths/total population	Troponin positive patients deaths/total deaths	Most common pathogen found in SRTE
Turner et al. [2]	15	12 (80%)	—	—	—		4 (26.7%)	4/15 (26.7%)	4/4 (100%)	—

Ammann et al. [3]*	20 (sepsis, SIRS, or septic shock) (no prior CAD ever)	17/20 (85%)	10/17 (58.8%)	1/17 (5.88%) CAD was found at autopsy	6/17 (35.2%)	10/11 (91%)	6 (30%)	5/20 (25%)	5/6 (83%)	53% gram positive, *Streptococcus pneumoniae*, 25% gram negative

Altmann et al. [4]*	38 (sepsis, SIRS, or septic shock) (no prior CAD ever)	22/38 (58%)	14/22 (64%)	1/22 CAD found at autopsy (4.54%)	7/22 (32%)	14/15 (93.33%)	10 (26%)	6/38 (15.7%)	6/10 (60%)	40% gram positive, 29% gram negative, 26% culture −ve, 5% fungal

Ammann et al. [5]*	58 (SIRS, sepsis, septic shock, and other diseases, 7 patients)	32/58 (55%) 32/51 (62.7%), 7 patients excluded as diagnosis was not SRTE	23/32 (72%)	2/32 (6%)	7 (22%)	23/25 (92%)	16 (28%)	13/51 (25.4%)	13/16 (81.3%)	Gram positive 29.4%, gram negative 37.2%, viral/fungal 0.078%, undetermined cause in remainder

Fernandes et al. [9]	10 (no prior CAD) (septic patients)	6	—	—	—	—	4/10 (40%)	3/10 (30%)	3/4 (75%)	—

Ver Elst et al. [10]	46 (septic shock patients) (some had prior history of CAD)	23 (50%)	—	—	—	—	21/46 (46%)	9/46 (19.5%)	9/21 (42.8%)	Gram negative bacteremia

Mehta et al. [11]	37 (sepsis, SIRS, or septic shock) (no prior CAD ever)	16/37 (43%)	—	—	—	—	15 (41%)	10/37 (27%)	10/15 (66.66%)	—

Arlati et al. [12]	19 septic shocks, severe sepsis patients	11/19 (58%)	—	—	—	—	10 (septic groups)	—	—	—

Spies et al. [13]	26	18 (69%)	—	—	—	—	18 (69%)	15 (58%)	15/18 (83%)	—

Summary of studies with the SRTE, *studies where some form of cardiac testing, invasive (angiogram) or noninvasive (stress echocardiogram), was done to rule out CAD in patients with no preexisting CAD, data from the autopsy results also included.
